# Effects of Droplet-Vitrification Cryopreservation Based on Physiological and Antioxidant Enzyme Activities of *Brassidium* Shooting Star Orchid

**DOI:** 10.1155/2015/961793

**Published:** 2015-03-11

**Authors:** Safrina Rahmah, Safiah Ahmad Mubbarakh, Khor Soo Ping, Sreeramanan Subramaniam

**Affiliations:** School of Biological Sciences, Universiti Sains Malaysia (USM), 11800 Penang, Malaysia

## Abstract

Protocorm-like bodies (PLBs) of *Brassidium* Shooting Star orchid were successfully cryopreserved using droplet-vitrification method. Vitrification based cryopreservation protocol is comprised of preculture, osmoprotection, cryoprotection, cooling, rewarming, and growth recovery and each and every step contributes to the achievement of successful cryopreservation. In order to reveal the lethal and nonlethal damage produced by cryopreservation, histological observation, scanning electron microscopy (SEM), and biochemical analysis were carried out in both cryopreserved and noncryopreserved PLBs of *Brassidium* Shooting Star orchid comparing with the control PLBs stock culture. Histological and scanning electron microscopy analyses displayed structural changes in cryopreserved PLBs due to the impact of cryoinjury during exposure to liquid nitrogen. Total soluble protein significantly increased throughout the dehydration process and the highest value was achieved when PLBs were stored in liquid nitrogen. Ascorbate peroxidase (APX) and catalase (CAT) showed the highest enzyme activities in both dehydration and cryostorage treatments indicating that stress level of PLBs was high during these stages.

## 1. Introduction

Conservation of orchid germplasm is important to protect biodiversity and also to store elite plants, the latter being necessary for the improvement and maintenance of new cultivars [1]. Orchidaceae belonging to 800 genera consists of almost 35,000 species and more than 150,000 artificial hybrids [2, 3]. A new orchid genus named* Brassidium *has been produced from the crosses of two genera* Brassia* and* Oncidium* .The unique colour combination of species and hybrids in these groups makes them as one of the popular traded groups with highly consumer demand [4]. Cryopreservation has become an important and valuable tool for long-term storage of germplasm via* ex situ* conservation. Cryopreservation refers to the process of freezing living materials at ultralow temperature of liquid nitrogen which allows the safe and cost effective long-term conservation for many plant cells and tissues [5, 6]. At this low temperature, all metabolic processes and cellular divisions were halted. Therefore, plant germplasm materials can be stored stably for unlimited durations [7].

Several factors which are involved in cryopreservation procedures promote stresses and cryoinjury to the plant. This includes changes in cellular structure, deterioration of the plasma membrane, and alteration of enzyme activity [8]. Water content is the critical factor in succesfull cryopreservation. Reducing water content from cells is important to avoid the phenomenon of ice crystallization. Cell damages commonly take place in osmoprotection and dehydration process as the cells do not withstand the osmotic stress when water is removed from protoplasm [9]. Besides that, cryoinjury induced by intracellular ice formation also promotes cell death during cooling and warming process. This is due to the uneven distribution of cryoprotectant throughout the cells, thus resulting in intracellular ice formation in several parts of the cells [10]. To study the effects of cryopreservation procedure to the plant, various analyses such as histological scanning electron microscopic observation and biochemical analysis can be done. Several numbers of histological studies using light microscopy have been reported to characterise and have better understanding of dehydration and freezing effect on cryopreserved cells [11, 12]. Scanning electron microscopy has also been carried out to observe the biological surface structure of the explants following cryopreservation [13–15].

In vitrification-based cryopreservation, plant materials were exposed to stress due to excision, dehydration, and changes in temperature during freezing and warming. Uncontrolled production of reactive oxygen species (ROS) was known as the main factor of causing reduction in cryopreserved explants regeneration and cell death after rewarming [16]. ROS are products of various normal cellular metabolism pathways in plants [17]. It comprises superoxide radicals, singlet oxygen, hydrogen peroxide, and hydroxyl radicals. They are highly reactive and toxic which can cause significant damage to cell components [18, 19]. Many abiotic stresses such as heavy metals, light intensity, drought, wounding, and extreme changes in temperature induce the overproduction of ROS. When production of ROS is higher than antioxidant in the cells, it results in oxidative stress and leads to cell death. To protect the cells against toxic effect of ROS, plants have developed natural antioxidant defence mechanisms [20]. ROS production is controlled by enzymatic antioxidant system such as superoxide dismutase (SOD), catalase (CAT), ascorbate peroxidase (APX), glutathione reductase (GR) and nonenzymatic antioxidative including ascorbic acid (ASH), glutathione (GSH), and *α*-tocopherols. If antioxidant defences are compromised, uncontrolled production of ROS will lead to oxidative damage to lipids, proteins, and DNA. Enzymatic antioxidants protect the cells by transforming ROS into stable inactive products and avoiding production of lipid peroxidation. On the other hand, nonenzymatic antioxidants act by scavenging ROS, stopping toxic byproducts of metal ions, and inhibiting the complex radical chain reactions [21]. Antioxidant supplements can increase the regeneration of some plant and algal cells from cryostorage [22, 23]. It has been used as freezing tolerance marker in the number of cryopreservation experiments [24–26].

Catalase (H_2_O_2_ oxidoreductase) which is a heme-containing enzyme catalyses the dismutation of H_2_O_2_ into H_2_O and O_2_ generated in peroxisomes during oxidative stress [27, 28]. Proliferation of peroxisomes during stresses aids in scavenging H_2_O_2_ diffusing from cytosol [29]. One molecule of catalase converts 6 million molecules of H_2_O_2_ to H_2_O and O_2_ and is said to have one of the highest turnover rates for all enzymes [19]. Besides reaction with H_2_O_2_, catalase also encounters hydroperoxidases such as methyl hydrogen peroxide (MeOOH) [30]. Catalase reduces H_2_O_2_ intensity in peroxisomes whereas APX carry out this role in chloroplast and cytosol of plant cells [31]. APX uses ascorbate as a hydrogen donor to break down H_2_O_2_ to form H_2_O and monodehydroascorbate (MDHA) [32]. APX has a higher affinity for H_2_O_2_ (*μ*M range) than CAT and POD (*μ*M range) and it may have a more essential role during stress [19]. The overproduction of APX amplified the POD activity which fortifies the ROS scavenging system and helps in oxidative stress tolerance [33]. Therefore, the objective of this study is to identify the effects of droplet vitrification cryopreservation procedure on PLBs of* Brassidium* Shooting Star orchid through histological analysis, SEM, and several biochemical assays (total soluble protein, APX, and CAT).

## 2. Materials and Methods

### 2.1. Planting Materials

*In vitro* culture of* Brassidium* Shooting Star orchid PLBs were initiated by aseptically culturing basal corms of orchid hybrid on half-strength MS media supplemented with 1 mg/L 6-benzylaminopurine (BAP), 2% sucrose, and 2.75 g/L Gelrite. Cultures were grown at 25 ± 2°C under 16-hour photoperiod and subcultured every three weeks.

### 2.2. Droplet Vitrification

For cryopreservation procedure, PLBs 3-4 mm in size were cultured in half-strength semisolid MS media supplemented with 0.25 M at 25 ± 2°C for 7 days under 16-hour photoperiod. PLBs were then treated with 4 mL of loading solution (osmoprotection) for 20 minutes at room temperature followed by dehydration with 4 mL of ice cooled PVS2 solution (cryoprotection) for 40 minutes. PLBs were then dropped with 15 *μ*L PVS2 solution on aluminium strips (5 × 20 mm) and plunged into liquid nitrogen. For rewarming, aluminium strips were taken out from the vial and quickly plunged into 4 mL unloading solution containing 1.2 M sucrose for 15 minutes at room temperature. PLBs were then transferred into half-strength MS media supplemented with 20 g/L sucrose and 2.75 g/L Gelrite, kept for two weeks in the dark condition, and then exposed to 16-hour photoperiod. Cryopreserved PLBs were transferred into half-strength semisolid MS media enriched with 1 mg/L BAP, 20 g/L sucrose, and 2.75 g/L Gelrite 4 weeks after cryotreatment.

### 2.3. Histological Analysis

In sample preparation for histological study, PLBs were fixed with formaldehyde (FAA) (95% ethyl alcohol : glacial acetic acid : formaldehyde : water, 10 : 1 : 2 : 7) for 48 h. After 24 h washing under running tap water, PLBs were then dehydrated with graded series of tertiary-butyl alcohol (TBA). After dehydration, PLBs were exposed to xylene for 10 minutes followed by combination of xylene and Shandon Histoplast Pelletised Paraffin Wax for 30 minutes at 60°C oven. Subsequently the samples were then treated with wax I, II, and III (increasing concentration of Shandon Histoplast Pelletised Paraffin Wax) for 1 hour, respectively, at 60°C oven. Specimen was then blocked by pouring melted Shandon Histoplast Pelletised Paraffin Wax into mould to form wax blocks and the samples were immediately positioned inside the wax block. The block was sliced with 10 Micron Microtome and the sliced ribbons were then attached to clean glass slide and kept in 40°C oven for 24 hours. Specimen was then stained with safranin and fast green. The observation of slides was conducted using light microscope.

### 2.4. Scanning Electron Microscope Analysis

PLBs were exposed to 2% osmium tetraoxide for vapour fixation process for 2 hours. Once the samples have been fixed, the planchette is plugged into slushy nitrogen (−210°C) and then transferred to the peltier-cooled stage of freeze dryer and left to freeze dry for 10 hours. The samples were spotted with about 5 to 10 nm of gold before viewing in scanning electron microscope.

### 2.5. Biochemical Analysis

In this study total soluble protein content, ascorbate peroxidise (APX) and catalase (CAT) enzymes activities were monitored separately after treatment of each cryopreservation step such as preculture, loading, PVS2, storage in LN, unloading, and recovery after 4 days of treatment. Fresh PLBs extracts were prepared at 4°C and activity assays were determined spectrophotometrically [34–37].

For total soluble protein content, about 1 g of PLBs was ground and homogenized in 3 mL of protein extraction buffer that contained 0.1 M Tris hydrochloric acid, 1.0 mM EDTA, and 0.1% mercaptoethanol (pH 8). The crude extracts were aliquoted into 1.5 mL microcentrifuge tubes and centrifuged at 12,000 rpm for 20 minutes (4°C). Total 20 *μ*L of PLB extract and 80 *μ*L of protein extraction buffer were added into test tube containing 5 mL of protein reagent (100 mg Coomassie Brilliant Blue G-250, 50 mL of 95% ethanol, and 100 mL of 85% (w/v) phosphoric acid were dissolved and topped up to 1L with double distilled water (pH 7.0)). The mixture was then incubated for 2 minutes and total protein content was measured using spectrophotometer at 595 nm. The protein content was compared with standard bovine serum albumin (BSA).

The protocol for enzyme extraction in APX assay was performed based on Elavarthi and Martin [38] method. About 200 mg of PLB was ground and homogenized in 1.2 mL of 0.2 M potassium phosphate buffer (pH 7.8 with 0.1 mM EDTA). The extracts were transferred into 1.5 mL microcentrifuge tubes and centrifuged at 13,000 rpm for 20 minutes. The supernatant was removed and the pellets were resuspended with 0.8 mL of the same buffer and centrifuged at 13,000 rpm for 15 minutes. APX activity was monitored using Nakano and Asada [35] method. The 3 mL assay contained 50 mM potassium phosphate buffer (pH 7.0), 0.5 mM ascorbate, 0.5 mM H_2_O_2_, and 30 *μ*L of PLB's supernatant obtained from each step of cryopreservation protocol. The reaction was initiated by adding H_2_O_2_ at last. The decrease of absorbance was monitored for every 30 seconds up to 3 minutes using a spectrophotometer set at 290 nm. The reduction of ascorbate was calculated using an extinction coefficient of 2.8 M^−1^·cm^−1^.

The protocol for enzyme extraction in CAT assay was performed based on Samantary [39] and Monnet et al. [37]. About 100 mg of PLB crude was homogenized in 10 mL of 100 mM potassium phosphate buffer (pH 7.8 with 2 mM EDTA ferric sodium and 2% polyvinylpyrrolidone), transferred into 50 mL centrifuge tubes, and centrifuged at 9000 rpm for 30 minutes. The quantification of CAT activity was determined using modified protocol used by Cakmak and Marschner [36] and Monnet et al. [37]. The 3.1 mL aliquot contained 100 mM potassium phosphate buffer (pH 7.0) and 100 *μ*L of PLB extract obtained from each step of cryopreservation protocol. The reaction was initiated by adding 6 mM H_2_O_2_. The decrease of absorbance was monitored for every 30 seconds up to 3 minutes using a spectrophotometer set at 240 nm. The decomposition of hydrogen peroxide was calculated using an extinction coefficient of 40 mM^−1^·cm^−1^.

The APX and CAT enzyme activities were expressed in unit of catalase (U·mL^−1^). Enzyme activities were calculated using Tijssen [40] and Flocco and Giuliettti [41] formula.

## 3. Results

### 3.1. Histology and Scanning Electron Microscopy of PLBs

**Figure 1:**
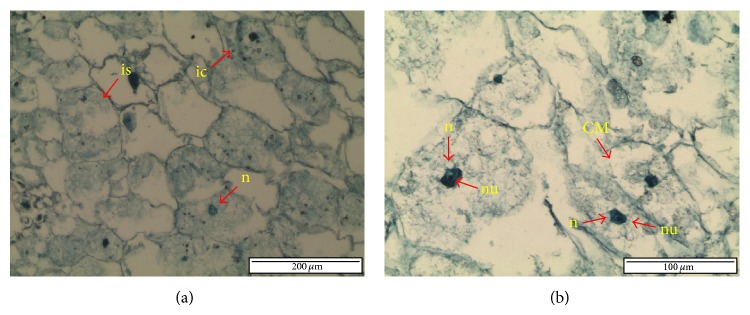
Cross-section of stock culture PLB. (a) The cells were intact with isodimetric shape and nucleus in central position. (b) Nucleus contains nucleolus and mitotic activities were observed (CM: cell mitosis; ic: intact cell; is: isodiametric; n: nucleus; nu: nucleolus).

Structural analysis of PLBs was observed using light and scanning electron microscopy. Cellular changes of stock culture and cryopreserved and noncryopreserved PLBs were monitored eight weeks after treatment. For histological analysis, cross-section of stock culture PLB showed that the shape of cells remained intact (Figure 1). There was no damage or cell plasmolysis observed, as cytoplasm was well preserved and displayed a very small periplasmic space. The densely stained cytoplasm contained a centrally positioned nucleus and clearly visible nucleoli. The mitosis activity was also noticed in the stock culture PLB.

**Figure 2:**
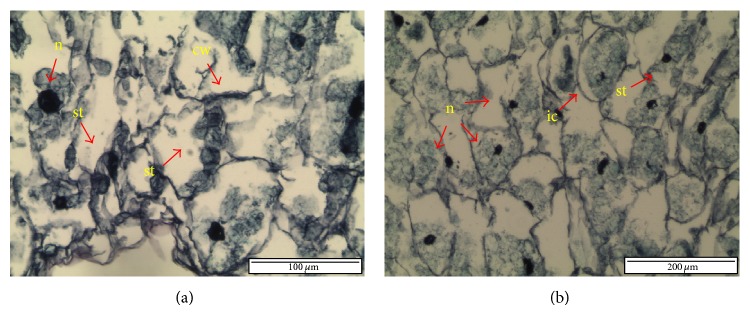
Cross-section of noncryopreserved PLB. (a) Original PLB. Nucleus was deeply stained, cell walls were thickening, and starch grains were dense. (b) Regenerated portion. Cells were intact with dense nuclei, and some starch was observed (ic: intact cell; cw: cell wall; n: nucleus; st: starch).

In this study, 73% regrowth was obtained when PLBs were treated with optimised cryopreservation protocols. PLBs that were precultured on 0.25 M sucrose for 7 days were treated with 20 minutes loading solution, followed by 40 minutes dehydration with PVS2 before plunging into liquid nitrogen. In noncryopreserved treatment, PLBs were directly rinsed with 1.2 M sucrose for 15 minutes after PVS2 dehydration treatment. Histological analysis revealed that dehydration process does not affect cellular structural of noncryopreserved PLBs (Figure 2). Cross-section of original and regenerated noncryopreserved PLB displayed intact cells, voluminous nucleus, and dense cytoplasm. Starch accumulation was clearly observed in both original and regenerated PLBs.

**Figure 3:**
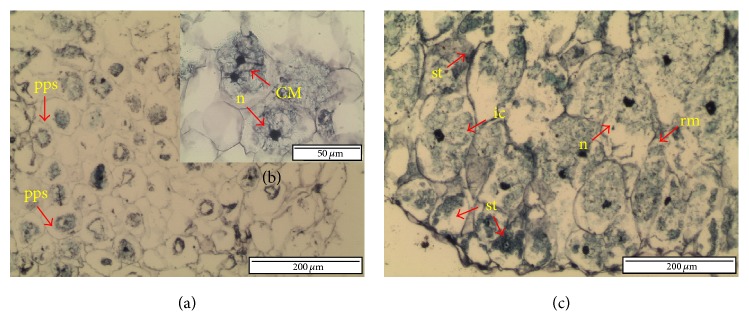
Cross-section of cryopreserved PLB. (a) Original PLB. Cells were in plasmolysis and pps were significant. (b) Higher magnification showing cell being intact and mitotic activity in cryopreserved PLB. (c) Regenerated portion. Cells were intact with dense nuclei, and some rupture membrane and starch were observed (CM: cell mitosis ic: intact cell; n: nucleus; pps: periplasmic space; rm: rupture membrane; st: starch).

Figure 3 displayed the effect of liquid nitrogen in cellular changes of PLB. Cross-section of cryopreserved PLB was less stained compared to the control. The cells experienced plasmolysis as cytoplasm shrunk and significant periplasmic spaces were noticed (Figure 3(a)). Nonetheless, mitotic activity was observed in the cryopreserved PLB (Figure 3(b)). This indicates that cryopreserved PLB successfully conserved living cells that would regenerate into new plantlets. However, cross-section of regenerated portion of cryopreserved PLB displayed structure similar to that of stock culture (Figure 3(c)). The PLBs were recovered with no damages and reduced periplasmic space. The nuclei were dense and highly stained. Starch accumulation was also visible in this PLB.

**Figure 4:**
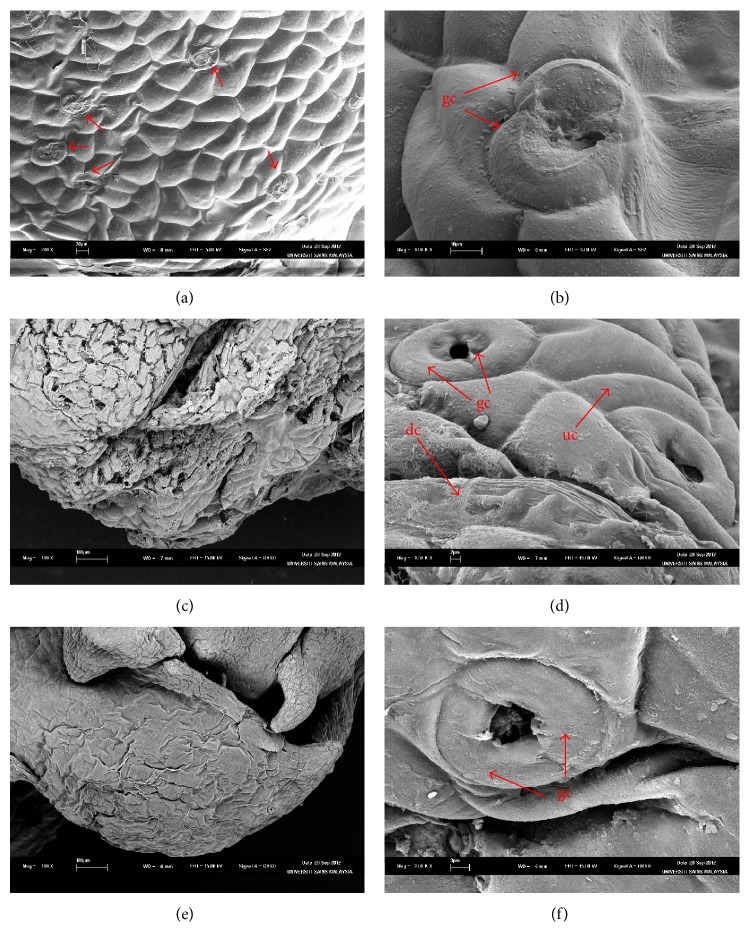
Scanning electron microscopic of stock culture, cryopreserved and noncryopreserved PLBs. (a) stock culture PLB with dense stomata on its surface (arrows), (b) higher magnification of stomata in stock culture PLB, (c) cryopreserved PLB showing damage, (d) higher magnification of cryopreserved PLB showing stomata and damage and undamaged cells, (e) noncryopreserved PLB shrunk, and (f) higher magnification showing stomata of noncryopreserved PLB (dg: damage cell; gc; guard cell; ud: undamaged cell).

SEM studies of both cryopreserved and noncryopreserved PLBs presented varying effects of the treatment using cryoprotectant and liquid nitrogen. Surface of both cryopreserved and noncryopreserved PLBs showed damage compared to stock culture (Figure 4). Stock culture PLB remain intact with randomly distributed stomata on the surface (Figure 4(a)). Contrarily, SEM analysis of cryopreserved PLB exhibited cracked on the surface (Figure 4(c)). However, highly magnified image of cryopreserved PLB (Figure 4(d)) showed the presence of intact cells indicating recovery of tissues and chances of regeneration. On the other hand, noncryopreserved PLB did not exhibit detrimental effects observed on the cryopreserved PLB. However, the noncryopreserved PLB shrunk indicating a minimal damage to the surface (Figure 4(e)). Cryopreserved and noncryopreserved PLBs were shown to be similar to the stock culture in terms of the occurrence of stomata. Both tissues exhibited randomly distributed oval-shaped stomata formed by intact guard cells (Figures 4(d) and 4(f)).

### 3.2. Biochemical Analyses

**Figure 5:**
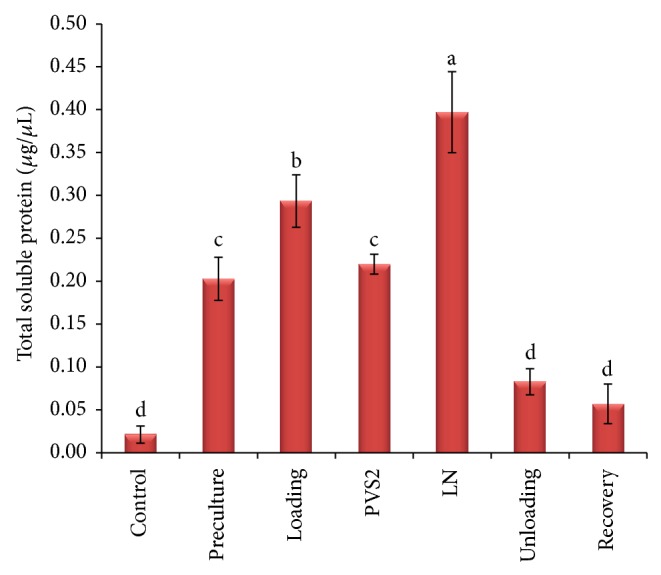
Total soluble protein contents of PLBs sampled at various stages of the cryopreservation. Results were analysed using one-way ANOVA. Means followed by the same alphabet were not significantly different using Tukey test.

**Figure 6:**
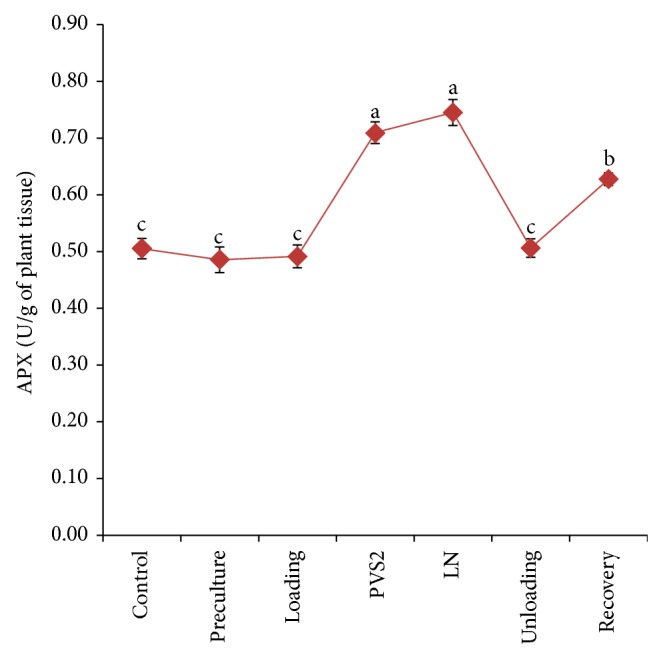
Changes in ascorbate peroxidase (APX) enzyme activity at various stages of the cryopreservation. Results were analysed using one-way ANOVA. Means followed by the same alphabet were not significantly different using Tukey test.

**Figure 7:**
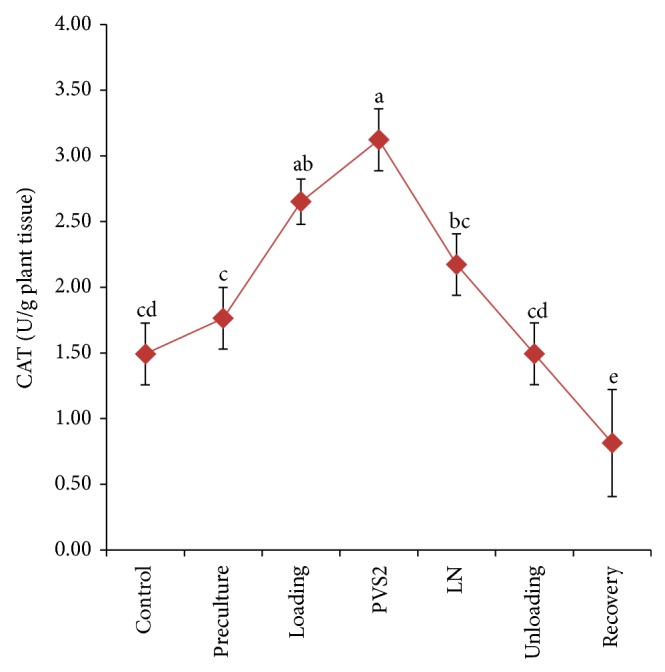
Changes in catalase (CAT) enzyme activity at various stages of the cryopreservation.Results were analysed using one-way ANOVA. Means followed by the same alphabet were not significantly different using Tukey test.

Cryopreservation procedures significantly affected the total soluble protein and enzyme activity of treated PLB (Figures 5–7). Increases in total soluble protein of PLBs were observed during early stage of cryopreservation, that is, preculture and loading treatment, when compared to control PLBs. There were slight decreases of total soluble protein content during PVS2 dehydration; however, the highest value was significantly achieved when PLBs were stored in liquid nitrogen (Figure 5). The total soluble protein content further decreased during unloading and recovery treatment and there were no significant differences compared to control. Catalase and ascorbate peroxidase displayed the highest antioxidant activity in both dehydration and cryostorage treatments (Figures 6 and 7). During preculture and loading treatment, APX activity is relatively low and did not show significant different with control (Figure 6). On the other hand, CAT activity represents a gradual increase from control to PVS2 dehydration treatment and subsequently decreases after liquid nitrogen storage until growth recovery stage (Figure 7).

## 4. Discussion

Excessive amount of water in cells may crystallize while lower amount of water causes cells to shrink during the event of cryopreservation of tissues. Hence, maintaining a balance water content in cells is the utmost factor to achieve sucessful cryopreservation of a tissue. Additional PVS2 solution was commonly used in vitrifcation-based cryopreservation for cell dehydration and changing behaviour of remaining water within the cells [42]. During cryopreservation procedures the damage will firstly occur in the cell membrane and results in cell death [10, 43]. Cryopreserved plant which successfully regenerated will show many survived cells in meristematic region [44].

PLBs made up of shoot apical meristem are able to regenerate into plantlets [45]. According to Volk and Caspersen [9], structural changes following cryopreservation allow the determination of cell sensitivity based on its type. Smaller cells such as meristem, upper cortex, and young leaf are less vulnerable to osmotic stress and plasmolysis during dehydration process. According to histological observation by N'Nan et al. [44], survival cell displayed isodiametric structure, dense cytoplasm with high nucleus-cytoplasm ratio, and spherical nucleus in a central position. Feng et al. [46] observed that the untreated cells showed dense staining and well-preserved cytoplasm in leaf primordial and apical dome. This is comparable with the present study, and the cross-section of stock culture PLB displayed intact cell with dense cytoplasm and small periplasmic space. Dense cytoplasm on the anterior of PLBs would continue to develop into a complete sheath leaf that would further enlarge and develop into true leaves [47].

In the present study, histological observation revealed that cell damage is mostly caused by cryoinjury during cooling and warming. Ineffective cryoprotection can be due to inhomogeneous tissue within the plant. Thus, several parts of the tissue can survive from cryopreservation through transformation into glassy state while other tissue parts do not survive as a result of ice formation and cell disintegration during rewarming [10]. In cryopreservation of orchid using PVS3 vitrification method, Mubbarakh et al. [45] reported the incidence of plasmolysis after cryopreservation. Cell membrane rupture with less dense cytoplasm, voluminous nucleus, increase in starch grains, and thickening of the cell wall was observed. The similar pattern of cell was observed in the present study. Cryopreserved cells displayed less stained, cytoplasm shrunk, and large periplasmic space (low nucleocytoplasm ratio) and highly stained nucleus (Figure 3(a)). Voluminous nucleus without a visible nucleolus suggested that nuclei experience osmotic stress [48]. Compact and deeply stained nucleus indicates that chromatin remains condensed and probably no transcription or protein synthesis for mitosis would be possible [11, 48]. In contrast to this study, the mitotic activity still exists in cryopreserved PLB although the nuclei are deeply stained (Figure 3(b)).

Angiosperms own a mechanism of resistance against dehydration where it builds up polysaccharide compounds in cells [49]. Survival of cell after thaw cryopreservation can be manipulated by optimising sucrose concentration and preculture duration [50]. During dehydration and chilling process, sugar stabilizes and maintains plasma lemma integrity by replacing water molecules and forms hydrogen bonding with the polar head group of membrane phospholipid. Therefore, cells without starch grains did not survive after cryopreservation [45]. According to Ganino et al. [51], sucrose treatment could increase starch accumulation and protect cell integrity, while exposure to PVS2 leads the cell to experience plasmolysis. Preculture of shoot tips on low sucrose media showed starch accumulation in meristematic cortex and leaf primordial [52–54], while preculture in higher sucrose concentration resulted in concave plasmolysis [9]. In the present study, noncryopreserved PLB displayed intact cells with starch accumulation and thickening of cell wall (Figure 2(a)). The cell walls are surrounded with proteins and enzymes that actively work to reform the wall during cell growth and prevent ruptures in the plasma membrane [55]. Likewise, it also thickens and strengthens during induced stress [56].

According to Poobathy et al. [15], cryoinjury did not occur at the exterior part of PLBs of* Dendrobium* sonia-28 as SEM observation of both cryopreserved and noncryopreserved PLBs showed intact epidermal layers. In contrast with this study, scanning electron micrograph of cryopreserved PLB showed damaging effect of cryopreservation procedures (Figure 4). Dehydration process caused PLB shrunk and cracks in cryopreserved PLB. Similar observations were reported in oil palm's polyembryonic and* Dendrobium *Bobby Messina's PLB [13, 14]. The damage in cell might result due to plasmolysis incidence which related based on histological analysis [57].

In this study, total soluble protein, ascorbate peroxidase, and catalase activities were measured as an indication of oxidative stress encountered by PLB of* Brassidium* Shooting Star during cryopreservation. Catalase and ascorbate peroxidases are part of antioxidant protection system enzymes which scavenge the reactive oxygen species in plants. These enzymes are largely confined to removal of hydrogen peroxide [58]. Increasing in protein expression was believed as one of the physiological responses against osmotic stress and may be induced by freezing tolerance mechanism [59, 60]. It has been suggested that plants under stress may build up such amount of proteins that are used as a source of storage of nitrogen which could be activated after stress relief [61]. Study of the effects of cryopreservation on protein expression in potato found that preculture treatment did not increase the protein content compared to the control [10]. Steady decrease in protein expression was observed after preculture step [10].

In contrast with the present study, total soluble protein in precultured PLBs showed drastically increased from control PLBs (Figure 5). However, increasing in catalase and ascorbate activity did not significantly observe in preculture treatment (Figures 6 and 7). This may be due to the accumulation of soluble sugar during preculture treatment in highly concentrated media, which may further change the protein metabolism to induce cell tolerance [62].

Most of the plant suffered from osmotic stress during osmoprotection and dehydration process. Wen et al. [63] observed that dehydration and freezing extensively increase APX activity in maize's embryo culture. Significant increase in catalase and ascorbate peroxidase activities after the PVS2 dehydration and cryostorage treatment may indicate excess production of superoxide as a result of osmotic and dehydration during cryopreservation [64]. On the other hand, decrease in catalase and peroxidase activities could result in accumulation of intracellular hydrogen peroxide and this phenomenon was correlated with tissue browning [65]. In contrast with enzyme activity, PVS2 treatment did not enhance the total soluble protein in PLBs. This is due to the vitrified state which was able to stabilize proteins in cells [66]. In the study on wheat species, Baek and Skinner [67] evaluated the expression of antioxidant enzyme such as SOD and catalase that increased after cold acclimation. Increasing of antioxidants expression in the plant provides the better tolerance to oxidative stress [68]. Study on genotypic tolerance of* Ribes* confirmed that more tolerant genotype exhibited higher level of antioxidant throughout recovery whereas sensitive genotype showed no difference in oxidative stress markers [25].

Microscopy and biochemical studies proved that during dehydration and storage in liquid nitrogen generated the most oxidative stress. Nevertheless, viability of PLBs was not closely related to changes in cellular structure but antioxidant enzyme activity. Therefore, to enhance the growth percentage of cryopreserved PLBs, additional studies should be made on the supplementation of exogenous antioxidants during cryopreservation stages.
